# Study on the Effect of PDCA Circulation Method on Nursing Quality Management in the Day Operating Room

**DOI:** 10.1155/2022/3503095

**Published:** 2022-05-12

**Authors:** Xiaoyan Yao, Hang Zhu, Yifei Wang, Yan Xiang, Ying Chen

**Affiliations:** ^1^Outpatient Department, The Second Hospital of Jiaxing, Jiaxing 314001, Zhejiang, China; ^2^Operating Room, The Second Hospital of Jiaxing, Jiaxing 314001, Zhejiang, China

## Abstract

**Objective:**

The main objective is to investigate the effect of PDCA circulation management on nursing quality in the day operation room.

**Methods:**

A retrospective study was performed in 300 patients in the day surgery room. For the control group, 150 patients received routine nursing. For the observation group, 150 patients underwent PDCA circulation nursing management. The scores for nursing quality management, the hospital infection, the detection rate of pathogenic bacteria, the incidence rate of adverse events, the negative emotion of patients, and the satisfaction rate for the day surgery department were recorded and analyzed between two groups.

**Results:**

Compared with the control group, the scores for nursing quality management and the satisfaction rate for the day surgery department were significantly increased (all *P* < 0.05), while the hospital infection, the detection rate of pathogenic bacteria, HAMA scores, HAMD scores, and the incidence rate of adverse events were obviously decreased (all *P* < 0.05). Significantly statistical differences were observed between the two groups.

**Conclusion:**

PDCA circulation nursing management in the day operating room could optimize the nursing quality management, improve the satisfaction rate of the operating room, reduce the negative emotions of patients, and prevent adverse events in time, with lower hospital infections.

## 1. Introduction

The operating room was the most important place for patients to receive the treatment of surgery. Compared with other departments, the operating room had higher requirements for the quality of care, due to the variability and high risk of work in the operating room [1, 2]. Operating room is an important place for surgical treatment. Its operation and quality management are directly related to the physical and mental health of patients and the surgical effect of doctors [3, 4]. The nursing work in the operating room ran through the entire operation. It was reported that there were some problems in the operative room such as the wide range of work in the operating room, the increase in the number of medical staff and the pressure of nursing work, an increase in the number of distractions in the work, and the risk of pollution and even infection. Therefore, taking effective nursing measures was of great significance to the success rate of surgery and the improvement of nursing quality.

At present, routine management was mainly applied in the operative room [5]. This method could assess the nursing quality and working status of nurses. However, it was reported that routine management usually leads to poor effects. As brand-new nursing management, PDCA circulation nursing management provided a more reasonable and high-quality service. Some studies reported that the performance of the PDCA circulation method in nursing work could really put nursing work into practice and improve nursing quality continuously and steadily [6–8]. In clinical practice, the nursing work in the day surgery room was confusing. And the prevention consciousness of these nurses was poor. All of these caused the poor quality of nursing management from the day surgical room.

In this context, 300 patients were enrolled in this study to investigate the performance of the PDCA circulation method in the nursing management of the day surgical room and its influence on nursing quality. The results of this study would guide the clinical nursing work in the day operating room.

## 2. Materials and Methods

### 2.1. General Information

In this study, 300 patients who underwent day surgery in the Second Hospital of Jiaxing from January 2019 to December 2020 were enrolled. Inclusion criteria were as follows: patients aged below 70 years; patients who had the indications for the day surgery; patients who signed the informed consent. Exclusion criteria were as follows: patients who were with severe liver and kidney dysfunction; patients who had cardiovascular and cerebrovascular diseases; patients who had severe infectious diseases; patients who lost consciousness; patients who had a mental illness; patients with incomplete medical records; patients who were unable to cooperate in this study.

This was a retrospective study. For the control group, 150 patients received routine nursing. For the observation group, 150 patients were treated with nursing using the PDCA circulation method. This study was approved by the Ethics Committee of the Second Hospital of Jiaxing.

### 2.2. Methods

Routine nursing in the operating room was performed in the control group. The details were as follows: obey and conduct the management procedures in the operating room, closely monitor the patient's vital signs, correctly place the position of the body, inform them of postoperative precautions, deal with nursing problems in time, and prepare the surgical supplies.

PDCA circulation method was performed in the observation group. (1) Plan: a quality control team was established, and the problems including unclear surgical site marking, wrong drug distribution, device management, and foreign body retention in the operating room were analyzed. The reasons for the above problems were investigated, and it was also judged whether they met management standards of the operating room and whether it was implemented according to the requirements. Pay attention to every detail of different problems from the operating room, set goals according to the actual situation, and develop solutions. (2) Implementation: according to the goals of nursing management, the itinerant nurses should visit the patients before the operation and inform them of the precautions before and after the operation. The case data and operation types should be checked before the operation, and then, patients enter the operating room. In the operating room, nurses assisted the patient to put the appropriate surgical position. The aseptic awareness should be strengthened during the operation. Monitor closely their vital signs and make emergency preparations. Strengthen the cooperation between the instrument nurses and the surgeons, pass skillfully the surgical instruments, and keep the surgical specimens. Count and mark the surgical instruments and materials after the operation. Pay attention to keeping the patient warm during the operation and remind the surgeons to shorten the time of skin disinfection as much as possible in order to reduce the exposure of the patient's skin. Answer patiently various questions from patients after surgery to eliminate their fear of disease. Organize regularly nursing staff to conduct the training for in-hospital professional knowledge and operation processes such as management, application, and maintenance of equipment from the operating room. Ensures the operating room environment is clean, and items are placed neatly. (3) Inspection: The nursing staff was encouraged to conduct self-inspection and mutual inspections. The head nurse conducted random inspections on the quality of the surgical department. The staff of the quality control office from the hospital evaluated various management tasks. Organizes regularly training for unqualified nursing staff to ensure the quality of care and improve their own workability and qualities. (4) Action: Conduct a detailed analysis of the staged management problems of nursing quality, find the solutions, further optimize various nursing processes in the operative room, and prevent nursing risks from occurring. Aim directly at developing the nursing strategies. Take the current problems as the next nursing goal to achieve circulation care.

### 2.3. Observed Indicators

The quality assessment for nursing management was in charge of the quality control group in the hospital. It included management of equipment, preparation of equipment, cooperation skills of itinerant nurses, disinfection and isolation, and quality and safety of nursing. Each item was 20 scores. The quality assessment for nursing management was recorded and analyzed between the two groups.

The negative emotion of patients was compared between the two groups. The negative emotion was evaluated by HAMA scores and HAMD scores. High scores indicated poor negative emotion. The adverse events including displacement of pipelines, damage to equipment, wrong placement of drugs, and drug extravasation were recorded and compared between the two groups. The incidence rate of hospital infection and the detectable rate of the pathogenic germs were compared between the two groups.

The assessment of satisfaction for the day surgery department was subjectively made according to the environment, sterilization management, nursing professionalism, working attitudes, placeable items, and nursing effects. The total score was 100 points, very satisfied (≥90 scores), satisfied (≤90 scores and ≥70 scores), and unsatisfied (≤70 scores).

### 2.4. Statistical Analysis

The collected data were analyzed using SPSS statistical software version 23.0 (IBM, USA). The measurement data were presented as mean ± standard deviation. *T* test was applied for intergroup comparison. The enumeration data were presented as number/percentage (*n*/%). A comparison between the two groups was performed using the chi-square test. The difference was statistically significant when *P* value was less than 0.05.

## 3. Results

### 3.1. Comparison of General Information

As seen in Table 1, there were no significant differences regarding age, gender, operative time, BMI, and underlying diseases between the two groups (all *P* > 0.05).

### 3.2. Comparison of the Scores for Nursing Management Quality

In the observation group, the scores for management of equipment, preparation of equipment, cooperation skills of itinerant nurses, disinfection and isolation, and quality and safety of nursing were 17.5 ± 2.3, 18.4 ± 2.10, 17.2 ± 1.8, 18.7 ± 1.6, and 19.2 ± 2.7, respectively. In the control group, the scores for management of equipment were 14.2 ± 1.9, the scores for preparation of equipment were 15.9 ± 1.4, and the scores for cooperation skills of itinerant nurses were 15.5 ± 1.6. The scores for disinfection and isolation were 16.1 ± 2.0, and the scores for quality and safety of nursing were 16.7 ± 1.3. Significant differences were observed in the scores of nursing management quality between the two groups (all *P* < 0.05), as shown in Table 2.

### 3.3. Comparison of the Negative Emotion after Nursing between the Two Groups

There was no statistical difference between HAMA scores and HAMD scores in the negative emotion before nursing between the two groups. After nursing, in the observation group, HAMA scores were 10.8 ± 1.9, and HAMD scores were 12.1 ± 2.5, which were lower than those in the control group. The statistical differences were found between the two groups (*P* < 0.05), as shown in Table 3.

### 3.4. Comparison of the Adverse Events

In the control group, there were 6 cases with a displacement of pipelines, 5 cases with damage to equipment, 7 cases with the wrong placement of drugs, and 7 cases with drug extravasation. The overall incidence rate of adverse events in the control group was 16.7%. In the observation group, there were 4 cases with the displacement of pipelines, 3 cases with damage to equipment, 3 cases with the wrong placement of drugs, and 2 cases with drug extravasation. The overall incidence rate of adverse events in the control group was 8.0%. A significant difference in the overall incidence rate of adverse events was found between the two groups (*P* < 0.05).

### 3.5. Comparison of Hospital Infection and Pathogens Detection

As shown in Table 4, the incidence rate of hospital infection in the observation group was 2.7%, which was significantly lower than that in the control group (6.0%). The detectable rate of Gram-positive bacteria and Gram-negative bacteria in the control group was 20% and 26.7%, respectively, which were obviously higher than those in the observation group. There were significant statistical differences between the two groups (all *P* < 0.05).

### 3.6. Comparison of Satisfaction for Day Surgery Department

The satisfaction rate for the day surgery department in the observation group was significantly higher than that in the control group (94.0% vs 81.3%, *P* < 0.05), as shown in Table 5.

## 4. Discussion

The quality of medical services had become an important pillar to ensure the sustainable development and long-term survival of hospitals. The management of nursing quality, as the most important part of the quality system of the medical service, played an important role in the process of hospital management. The quality of nursing quality management directly affects the quality of medical services and social image. The day operative room was an important place in the hospital and assumed responsibility for the surgical treatment of patients. There were high medical risks and heavy pressure of work in the day operating room. The quality of nursing work in the day operating room was directly related to the prognosis and safety of patients, which indicated that it was very critical to implement good nursing quality management in the operating room [9, 10]. Therefore, choosing a scientific nursing management method had been an important topic for the department of nursing management paying attention.

In the nursing quality management of the day operative room, the traditional management model was not scientific, resulting in an inability to motivate the enthusiasm for nursing work. In addition, special conditions in the nursing management of the day operating room included a large flow of patients and a shortage of nurses. The routine nursing management could not meet the demands of modern medical services. PDCA circulation method was first proposed by Dr. Deming in the 1950s [8, 11]. PDCA cycle management method, as a new quality management model, was divided into four parts, such as planning part, implementation part, inspection part, and treatment part. These four parts usually are performed successively or at the same time [12, 13]. PDCA circulation method was suitable for different types of management and considered as an effective method to enhance the internal management. It was shown that this management method emphasized the circulation of management, and through the PDCA circulation method, it could organize various nursing works organically in the departments and ultimately ensure the overall quality of nursing [14]. Some studies reported that the PDCA circulation method was applied in the operating room, which could gradually find and solve the problems in the process of nursing management in the operating room through the four steps including planning, implementation, inspection, and treatment, so as to make the management of the operating room proceed toward the sounder and better direction [15]. Results of the current study showed that the scores for nursing management quality including management of equipment, preparation of equipment, cooperation skills of itinerant nurses, disinfection and isolation, and quality and safety of nursing were higher in the observation group. According to the comparison of the adverse events existing in the quality management of nursing, the incidence rates of displacement of pipelines, damage to equipment, wrong placement of drugs, and drug extravasation were lower in the observation group. The satisfactory rate and negative emotional conditions of patients not only reflected the dynamic change in the nursing process during hospitalization but also were the basis for assessing the professional ability of nurses and nursing quality management. In this study, HAMA scores and HAMD scores were significantly lower, and the satisfactory rate was obviously higher in the observation group. As we can see, via the performance of the PDCA circulation method, the nursing quality of management in the day operation room was effectively improved, and problems of nursing work were further decreased.

Moreover, hospital infection was an important step for controlling the overall infection in the hospital. The measures for enhanced control of intraoperative infection may involve extensive areas, such as high-concentration oxygen inhalation, control of perioperative temperature, blood sugar control, and the use of antibiotics. It was also reported that errors in the various parts such as operation specification of medical staff in the day operating room, the cleaning and disinfection of surgical instruments, the air quality of the operating room, and the movement of personnel were the important factors for resulting in local and systemic infections [16]. The results of this study showed that the incidences of hospital infections and the detective rate of pathogens in the observation group were significantly lower than that in the control group. It was because the nursing quality related to infection in the day operating room was increased in the process of repeated analysis and improvement. The application of PDCA cycle management in the prevention and control of infection in the day operating room could objectively, scientifically, and intuitively evaluate the indicators that were likely to cause infection. It was found that the advantages of the PDCA cycle were based on the big ring covering the small ring, which could greatly increase the execution efficiency of the work and further improve the nursing quality of the day operation room [17].

## 5. Conclusion

In conclusion, the performance of the PDCA circulation method in the nursing management of the day operating room could effectively improve the quality and safety of nursing, alleviate the negative emotions of patients, and elevate the satisfaction of patients. This new model of PDCA cycle nursing management is worth promoting clinically.

## Figures and Tables

**Table 1:** Comparison of basic data between two groups.

Group	Control group(*n* = 150)	Observation group(*n* = 150)
Age (years)	43.6 ± 5.7	45.3 ± 4.9
Gender (*n*)		
Male	95	101
Female	65	54
Operative time (h)	1.6 ± 0.5	1.4 ± 0.6
BMI (kg/m^2^)	21.6 ± 1.3	20.9 ± 1.1
Underlying disease		
Hypertension (*n*)	47	51
Diabetes (*n*)	42	38

**Table 2:** Comparison of the scores for nursing management quality between two groups.

Groups	Management of equipment	Preparation of equipment	Cooperation skills of itinerant nurses	Disinfection and isolation	Quality and safety of nursing
Control group (*N* = 150)	14.2 ± 1.9	15.9 ± 1.4	15.5 ± 1.6	16.1 ± 2.0	16.7 ± 1.3

Observation group (*N* = 150)	17.5 ± 2.3^*∗*^	18.4 ± 2.10^*∗*^	17.2 ± 1.8^*∗*^	18.7 ± 1.6^*∗*^	19.2 ± 2.7^*∗*^

*Note.* ^*∗*^indicates that there was a significant difference between the two groups.

**Table 3:** Comparison of HAMA scores and HAMD scores after nursing between the two groups.

Groups	HAMA scores	HAMD scores
Control group	16.1 ± 2.8	16.9 ± 3.1
Observation group	10.8 ± 1.9^*∗*^	12.1 ± 2.5^*∗*^

*Note.* ^*∗*^indicates that there was a significant difference between the two groups.

**Table 4:** Comparison of the incidence rate of hospital infection and the detectable rate of the pathogenic germs between the two groups.

Groups	Hospital infection (*N* (%))			Pathogens detection (*N* (%))	
	Respiratory tract infection	Urinary tract infection	Wound infection	Gram-positive bacteria	Gram-negative bacteria
Control group (*N* = 150)	4 (2.7%)	2 (1.3%)	3 (2.0%)	30 (20%)	40 (26.7%)

Observation group (*N* = 150)	2 (1.3%)	1 (0.7%)	1 (0.7%)	7 (4.7%)^*∗*^	11 (7.3%)^*∗*^

*Note.* ^*∗*^indicates that there was a significant difference between the two groups.

**Table 5:** Comparison of the satisfaction rate for day surgery department between two groups.

Group	Very satisfied	Satisfied	Unsatisfied	Very satisfied and satisfied
Control group	56	66	28	81.3%
Observation group	81	60	9	94.0%^*∗*^

*Note.* ^*∗*^indicates that there was a significant difference between the two groups.

## Data Availability

The raw data supporting the conclusions of this article are available by the authors, without undue reservation.
